# Application of Chitosan-Based Hydrogel Obtained from Insects in Pine Planting

**DOI:** 10.1155/2023/8175405

**Published:** 2023-03-28

**Authors:** Yerlan Zhatkanbayev, Zhanna Zhatkanbayeva, Zhanar Iskakova, Ainagul Kolpek, Almas Serikov, Nazira Moldagulova, Gaziza Danlybayeva, Ainur Sarsenova, Sandugash Anuarbekova

**Affiliations:** ^1^Faculty of Technology, Kazakh University of Technology and Business, Nur-Sultan 010000, K. Mukhamedkhanova 37 A Str, Kazakhstan; ^2^Research Institute of New Chemical Technologies, L.N. Gumilyov Eurasian National University, Nur-Sultan 010008, Satpaev 2 Str, Kazakhstan; ^3^LLP “Ecostandart.kz”, Nur-Sultan 010000, Sh.Valikhanov 13/1 Str, Kazakhstan

## Abstract

Agrogels, a hydrogel applied in the soil that collects water during irrigation or rainfall and distributes moisture to plant roots during drought, are a solution to water shortage concerns. Extending the release of low molecular weight chemicals has the potential to minimise mineral fertiliser losses as well as water and soil pollution. Thus, the aim of the research is to obtain chitosan from insect chitin, to synthesize a hydrogel based on chitosan with included mineral and organic fertilisers, and to report on experiments with agrogels in the field. In this study, chitosan was obtained from the adult beetles *Zophobas morio*. IR spectroscopy was used to examine chitosan. The existence of absorption lines typical of primary amines was demonstrated. In one step, a technique for the manufacture of chitosan-based hydrogels containing embedded mineral fertilisers was established. Hydrogel has a swelling coefficient of 60 g/g. Agrogels were evaluated while planting spruce seedlings on “Semei Ormany” LLP experimental locations. The survival rate of seedlings was found to be 40% higher in the experimental group than in the control group.

## 1. Introduction

Harsh continental climate Kazakhstan's territory defines the presence of arid regions, therefore, the dependence of the agricultural harvesting on weather conditions, which negatively affects soil productivity and increases the consumption of water for irrigation. Plants require a constant supply of water and mineral fertiliser application in order to function normally. Synthetic hydrogels capable of absorbing a large volume of water and then gradually releasing it are currently used to solve water supply problems in arid regions [1, 2]. As a result, it is necessary to create a hydrogel capable of decomposing under natural conditions without the formation of harmful substances, and chitosan is one such compound. Plants require mineral fertilisers in addition to water for nutrition [3]. Iontoponics technology is used to apply and extend the supply of mineral fertilisers. It is the process of introducing an ion-exchange resin (ionite) based on crosslinked polystyrene into the soil. When irrigated, the ion-exchange resin releases fertiliser into water; however, once the ion reserves are depleted, the ion-exchange resin becomes a soil pollutant [3, 4], as it is no longer suitable for regeneration and biodegradation.

Numerous studies on the synthesis of chitosan-based hydrogels have previously been conducted [5], but the process of embedding mineral fertilisers by sorption is a time-consuming and energy-consuming process due to the slow sorption of fertiliser ions from an aqueous solution and subsequent drying of the swollen hydrogel. Hydrogel swelling can reach up to 50 g of water per 1 g of dry polymer [6]. The use of agrogels will save water and nutrients in the root layer, as well as prolong fertiliser release, resulting in significant reductions in consumption and pollution of the environment.

The synthesis of biodegradable hydrogels with embedded plant nutrients will allow desert lands to be used for agriculture without contaminating the soil. The use of hydrogels based on chitosan extracted from the shells of crabs, shrimp, and other animals for various purposes is frequently found in the scientific literature. The lack of traditional sources of chitin in Kazakhstan drives up the price of chitosan. Insects, in addition to shell inhabitants of the sea, possess chitinous shells. The industrial production of such insects will provide Kazakhstan and other countries with low-cost chitosan.

In this article, chitinous shell insects are considered as an alternative source of chitosan. After death, *Zophobas morio* larvae and adult beetles used as breeding stock in industrial production become biological waste. At the same time, waste utilisation and extraction of such a useful component as chitin allows for a reduction in environmental load while obtaining an important and scarce component. Thus, the goal was to create a hydrogel based on natural compounds that could be further degraded (destroyed) in a natural way without harming nature and, in some cases, even bringing useful components.

The goal of this research is to test a biodegradable chitosan-based hydrogel derived from insect chitin, as well as to develop technology for the one-step synthesis of hydrogel with embedded mineral fertilisers.

## 2. Materials and Methodology

### 2.1. Experimental Site Conditions

The study's focus is on hydrogels made from chitosan derived from adult beetles *Zophobas morio*.

1 M NaOH at a solid/liquid ratio of 1 : 10 (g/ml) was added to a sample of adult beetles diced to less than 1 mm in size for deproteinization. The reaction was carried out under constant stirring at 80°C for 3 hours. The substance was filtered and rinsed with distilled water to neutralise the pH after 3 hours. The chitin was then bleached by immersing it in a 20% hydrogen peroxide solution for 10 minutes, and it was dried at 70°C following washing. Deacetylation of chitin was performed by treating it with NaOH solution with a concentration of 100 g/ml at a solid/liquid ratio = 1 : 10 (g/ml). The reaction mixture was incubated at 80°C for 16 hours stirring constantly at a speed of 250 rpm. The obtained chitosan was filtered, washed with distilled water to neutral pH, and dried at 70°C to constant mass.

### 2.2. Chitosan-Based Hydrogel Synthesis with Embedded Fertilisers

A number of studies were carried out in order to create chitosan-based hydrogels. Chitosan was dissolved in aqueous lactic or phosphoric acid solutions. The same solution was then treated with a suspension of one of the mineral fertilisers-ammonium nitrate, sodium hydrophosphate, or sodium humates. Afterward, a solution of glutaric aldehyde was added to the solution, and the reaction mixture was heated at temperatures from 40°C to 90°C for 2–24 hours. The hydrogels were rinsed 2-3 times with distilled water and dried to constant mass when the synthesis time was up. The swelling degree and gel fraction yield were then determined. Featured in a patent [7].

The hydrogels were tested in the “Semey Ormany” experimental grounds in eastern Kazakhstan.

### 2.3. The Experimental Locations' Soil Qualities and Composition

Sandy soils are in their primitive state. The humus horizon ranges in thickness from 1–3 to 10–15 cm. A water table emerges above the underlying rocks when soils build on two-member sandy-clay deposits. The ferruginous horizon forms in the capillary zone, whereas the gley horizon forms in water-saturated sand. Red-brown ferruginous interlayers can be seen in undeveined or old rewetted sands under pine forests. When dry, the humus horizon is loose or poorly compacted, unstructured, and free-flowing. Humus level ranges from 0.3 to 0.6%, whereas nitrogen content ranges from 0.02 to 0.05%. The reactivity of the water extract changes from mildly acidic to alkaline (pH 6.1–8.1) in the upper half-meter layer, and from highly acidic to strongly alkaline in the underlying rocks. The presence of organic matter in primitive soils and the depth of groundwater are both important factors in afforestation success. Pine trees can take root quickly if the water table depth is 70–100 cm, but they are not stable: in dry years, they can shrink due to a sharp decrease in water level and lack of moisture, and in wet years, they can shrink due to soaking of root systems. Plantations become more stable as the roots develop a sandy thickness above the groundwater table.

The soil profile is distinguished by the low thickness of humus horizons (A + B), which ranges from 30 to 40 cm, and their significant detritalization, which increases with depth and in the very surface layer. Humus concentration ranges between 0.7 and 15%. Soils in the upper portion of the humus layers are grayish-brown or grey, changing to light brown or brown as they descend. There is a horizon of carbonate buildup in the lower or somewhat lower regions of the humus layers, with evident excretions in the form of crusts and swellings on the bottom surfaces of debris. The higher half of the humus horizon has a friable structure, whereas the lower section is dusty and cloddy, occasionally nutty, and cloddy. Light and medium loamy cultivars predominate in terms of mechanical composition.

### 2.4. Climatic Conditions of the Sites

The climate in East Kazakhstan is harsh continental. The continentality of the climate is characterised by sharp temperature fluctuations, dry air, and a low amount of precipitation over the majority of the territory. The location of the reserve territory almost in the centre of the Asian continent, the manifestation of latitudinal and vertical climatic zoning in connection with the considerable length from north to south, large variations in absolute altitude of the area, and the features of the orography all influence the diversity and features of the climate.

The average annual amount of atmospheric precipitation here is up to 400–600, and during the warm period, it falls down to 70–80%, and during July, it is up to 25–30% of the annual amount. The height of snow cover reaches 50 cm by the end of winter, and the depth of soil frost is about 60 cm on average. The average annual air temperature is 2.9°C. The warmest month of the year is July (19–20°C), and the coldest is January (−16–18°C). The average duration of the warm period is 200–210 days and the frostless period is 115–120 days. There is rather intensive wind activity, which intensifies in the winter-spring period (in the spring-summer period thundershowers (8–10 cases) are observed here, especially during rainfalls of rainfall character).

The wind and poplar forests are located in the Irtysh River floodplain. There is a high floodplain terrace in some places, primarily in the lower section of the floodplain, that rises up to 3–5 meters above the low-water table and is flooded very rarely, only in years with a large flood. The terrace is typically made of clay saline rocks.

The climate for the growth of biocenosis is harsh; in summer, the evaporation rate far exceeds the amount of precipitation, indicating aridity. Dry, dusty weather dries out the soil, dehydrates plants, and traps them in soil particles.

## 3. Result and Discussion

The thick layer of insect carapace is known to be composed of chitin fibres embedded in a protein matrix [8]. Chitin content can reach up to 50% in some insect species [9]. In the current study, samples of *Zophobas morio*, a dunlin family beetle, were taken for chitin extraction using a sequential deprotection and deacetylation procedure. The trials employed dry remains of adults (imago) crushed to a size of less than 1 mm. Chitin and chitosan were produced as a result of deproteinization and deacetylation, respectively. The chitosan throughput yield was 15–18% of the dry weight of the beetles, whose functional composition was evaluated using infrared spectroscopy. The IR-spectra of the samples recorded vibrations characteristic of C=O carbonyl groups, esters (1762 cm^−1^), -amines (3127 cm^−1^), -aliphatic groups -CH (2900, 2800 cm^−1^), -C-C aromatic hydrocarbons (1602 cm^−1^), and -high-molecular polycyclic aromatic compounds (825, 722 cm^−1^) [10].

The synthesis of chitosan-based hydrogels has been extensively researched and presented in multiple papers. Most chitosan hydrogels are created by interacting with a glutaric aldehyde in acid media at temperatures ranging from 60°C to 100°C, as well as other bifunctional chemicals. Mineral fertilisers were also integrated into the hydrogel structure throughout the manufacturing process. The addition of nitrate in the flesh to the saturation limit of the solution, for example, has no effect on the development of hydrogel. Phosphates cannot be impeded in substantial amounts because of their limited solubility in the acidic aqueous solution of chitosan. Phosphates can be inhibited in high quantities by dissolving chitosan in phosphoric acid, followed by neutralisation. Organic acids (lactic acid) have no influence on the cross-linking process of chitosan. The following study findings are based on the usage of lactic acid as an acidic agent. The hydrogel production exceeded 80% of the original chitosan yield. The hydrogel swelled at a rate of 60 g of water per gram of dry polymer. The ideal synthesis conditions were determined; the synthesis of the hydrogel is detailed in further detail in the preceding patent. Thus, chitosan was extracted from the adult beetles *Zophobas morio* in this study. Chitosan was studied using infrared spectroscopy. In one step, a hydrogel containing embedded mineral fertilisers was created. The hydrogel has a swelling rate of 60 g/g.

Water, which swiftly filters through the sand and escapes into subterranean reservoirs at depths of 10 to 100 meters, prevents vegetation from growing adequately in deserts. However, in deserts, there are other suitable conditions-high insolation (a lot of solar radiation), the temperature spectrum of 20 to 500°C, and the absence of pests. The main problem of growing in deserts is water retention in the upper layer (10–20 cm).

When hydrogel particles are uniformly distributed in the substrate and supplied to the root layer, they are positioned in the pores and expand when moisture enters, producing an increase in moisture compared to untreated soil and creating ideal circumstances for plant development. The root system has access to water that has been concentrated in the hydrogel. To draw water, the plant's rhizome enters the agrogel's body.

Seedling rhizomes were treated with swelling finely powdered hydrogel plot #1, a combination of hydrogel and clay suspension plot #2, and simply clay suspension as a control measure. Each treatment was tried on two hectares of land. The engraftment parameter, i.e., the ratio of engrafted seedlings to the total number of seedlings seeded, was regulated. The experiment's conditions are listed.

One of the main objectives of RGU GLPR “Semei Ormany” is the reproduction of forest on fallow lands. For forest planting, 2-year-old seedlings of common pine and seedlings with closed root systems are grown.

Planting occurs in early spring when the snow cover melts, and within a short agrotechnical period (10–15 days), as well as in October, when seedlings have closed root systems. Planting is made, according to the project, the scheme of planting is 0.7 × 3.0 meters. On one hectare 4760 seedlings are planted. MPP-1 and SLN-1 forest planting machines are used for planting. Machines for planting trees MPP-1 plants trees in furrows without preparing the soil. Furrows are pre-cut for SLN-1 planting equipment. The thickness of the trunk at the root neck of Scots pine at the age of two years must be at least 2.5 mm, the height of the above-ground section of the seedling must be 12–15 cm, and the root system must be 25–30 cm.

**Figure 1:**
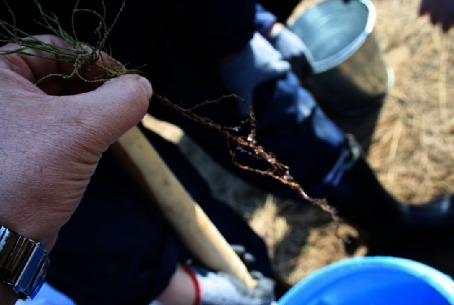
Method of planting-application of hydrogel suspension to the roots of pine seedlings.

The suburban forestry division of RGU GLPR “Semei Ormany” in Semipalatinsk carried out mechanical planting of two-year-old Scots pine seedlings with hydrogel. Previously, to keep seedling roots from drying out during planting and early development, the roots were dipped in a thick clay solution. Clay, on the other hand, has a poor water capacity. We replaced it entirely or partially with swelling hydrogel (Figure 1).

Agrogel is a polymer granule, which may also be manufactured as a powder, that swells and becomes gel-like when diluted with water. When you apply hydrogel to the root zone of plants, it continually supplies the plant with the proper amount of water. Changes in soil moisture do not stress the plant. 1 g of agrogel may hold 50–300 ml of water. Agrogel is absolutely harmless to both plants and people.

**Figure 2:**
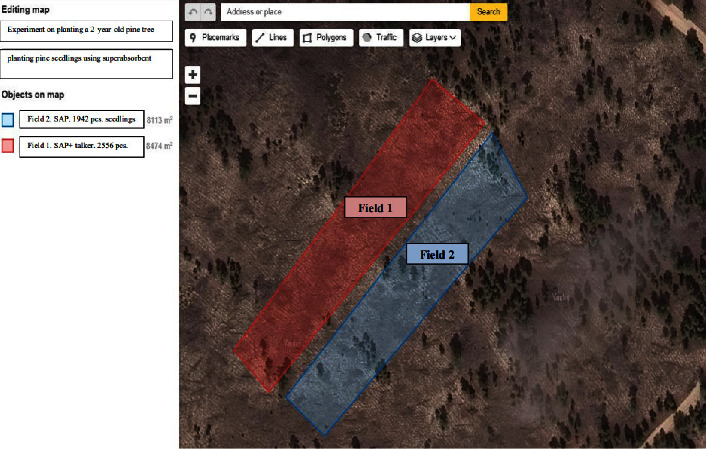
Google map location of trial plots 1 and 2.

The experimental plot consists of 2 experimental fields located in the suburban forestry Semipalatinsk branch of “Semei Ormany” in quarter 104 with coordinates of experimental field #1 (area 8474 m^2^), Google Map photo of experimental fields location is presented in Figure 2.

  Point 1: N = 50°27′11.923; E = 80°28′09.845
  Point 2: N = 50°27′12.814; E = 80°28′08.559
  Point 3: N = 50°27′18.057; E = 80°28′15.496
  Point 4: N = 50°27′17.240; E = 80°28′17.362
  Coordinates of the experimental field No. 2 (area of the field is 8113 m^2^)
  Point 1: N = 50°27′11.123; E = 80°28′11.830
  Point 2: N = 50°27′11.739; E = 80°28′10.634
  Point 3: N = 50°27′17.257; E = 80°28′17.360
  Point 4: N = 50°27′15.732; E = 80°28′18.789

Scots pine seedlings are 2 years old. The above-ground height of the seedlings ranges from 7 cm to 15 cm, and the root system is 25–30 cm long. The planting material was dug into the earth before being covered with snow and straw on top.

On field #1 (8474 m^2^), 2556 seedlings were planted. For 30 liters of water, 600 g of dry hydrogel was dissolved and 25 kg of clay and ∼10 liters of water were added. The solution was stirred until a homogeneous mass with a consistency similar to liquid sour cream. Seedling root systems in bundles of 50–60 pcs were dipped into the resulting solution and placed in boxes for planting. Furthermore, mechanized planting was carried out on the allocated plots.

On field #2 (8113 m^2^), 1942 seedlings were planted. Seedlings were dipped in agro gel solution only. To obtain the solution, 1 kg of dry gel was dissolved in 40 liters of water.

The consumption of dry hydrogel with the “hydrogel + clay” planting technique is 0.5 kg per 2000 seedlings. The usage of dry gel is 1 kg per 2000 seedlings when just “hydrogel” is used for planting. The solutions adhere nicely to the roots of seedlings in both techniques.

Because trees were already growing in the designated plots, the number of seedlings in fields 1 and 2 was different, 2,556 and 1,942, respectively. If the field is perfectly clean and level, up to 4,760 saplings can be planted on 1 hectare (10,000 m^2^).

A forest crop inventory count is described as the availability of forest crops, their area, and condition as determined by field examination. The inventory is carried out in the fall, at the conclusion of the vegetative season of plants, in various terms depending on the forest zone, but no sooner than September 1st and no later than October 15th. Only live plants with surviving healthy apical shoots in conifers and deciduous wood-with the potential to continue growing from a dormant bud-are considered during the inventory.

**Figure 3:**
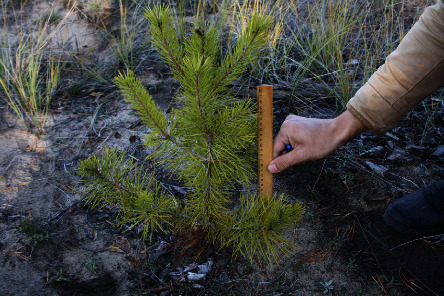
Photo of pine trees in plot 1 in the second year after planting.

As part of the experiment, based on the results of the first month, an inventory of seedlings was carried out in a month. The survival rate of seedlings on the experimental plots is 98 ± 1%.  Figure 3 shows a photo of a pine at the experimental site number 1.

Following a seasonal inventory of Scots pine seedlings in the fall, spring, and autumn of the following year.  Table 1 displays inventory statistics.

In conifers, only live plants with surviving healthy apical shoots are counted in the inventory. According to the findings of the studies, the use of hydrogel when planting forest crops has a good influence on plant survival.

Calculations and experiments have shown that roots can extract up to 95% of the water from gel; this is also confirmed in Salem et al. [11]. The importance of hydrogel additives in soil water balance is a partial transfer of moisture from gravitational flow into the accumulated soil state because hydrogel moisture is their modynamically closer to capillary moisture.

According to Kazansky et al. [12], the use of polymeric hydrogel increased the available moisture stock of sand by 2.4–8 times while decreasing water losses due to evaporation. The impediment of polyacrylamide type hydrogel in doses of 0.002–0.04% (100 kg/ha–2000 kg/ha) in the sand increased its moisture content from 1 to 20% in studies Benkenstein et al. [13]. In some cases, the biological effect of polymeric hydrogel application was discovered at such low doses (up to 0.001%) [14].

The decrease in soil density caused by hydrogel application created additional porosity, increasing water holding capacity to 41.7–43.5% compared to 23.8% in the control [15].

According to the results of the last inventory of seedlings in October 2020, the survival rate of seedlings in the experimental plots were as follows:

- In field #1 viable plants with preserved healthy apical shoots in coniferous crops −38.5% of the number of seedlings planted
- On the field No. 2 viable crops −40.9% of the number of seedlings planted at the given site
- In the control plot, where hydrogel was not used for planting −21.6%

## 4. Conclusion

Finally, chitosan was extracted from the adult beetles *Zophobas morio*. IR spectroscopy was used to examine chitosan. The existence of absorption lines typical of primary amines was demonstrated. In one step, a technique for the manufacture of chitosan-based hydrogels containing embedded mineral fertilisers was established. The introduction of ammonium nitrate, phosphates, lactic acid, and humates in suspension has been established. Hydrogel has a swelling coefficient of 60 g/g.

The hydrogel was tested in the field on the survival rate of two-year Scots pine seedlings in a quarter No .104 of the Semipalatinsk State Forestry Enterprise's suburban forestry Semipalatinsk branch.

The adaptability of seedlings was 38.5% when a mixture of hydrogel and clay was used, and 40.9% when only hydrogel was used. In comparison, when no hydrogel was applied, adaptability was 21.6%. The number of engrafted seedlings rose nearly twofold when the hydrogel was used compared to when it was not used.

As a result, a technology for producing biodegradable hydrogels containing fertilisers is proposed. In this case, the hydrogel is made from chitosan obtained from insects. Residents of areas without access to the sea can use this technology. The efficiency of using hydrogel when planting pine seedlings increased nearly twice that of not using hydrogel.

## Figures and Tables

**Table 1:** Inventory of pine seedling.

Site	Quantity (pcs)	Hydrogel consumption per 2000 seedlings	Adaptability (%)		
			September 2019	April 2020	October 2020
No. 1	2526	0.5 kg, hydrogel + clay	43.4 ± 0.14	40.2 ± 0.32	38.5 ± 0.26
No. 2	1942	1 kg, hydrogel	47.4 ± 0.21	42.7 ± 0.12	40.9 ± 0.4
Control	1631	Clay	27.8 ± 0.13	22.6 ± 0.17	21.6 ± 0.7

## Data Availability

Data is available.
